# Melt- vs. Non-Melt Blending of Complexly Processable Ultra-High Molecular Weight Polyethylene/Cellulose Nanofiber Bionanocomposite

**DOI:** 10.3390/polym13030404

**Published:** 2021-01-27

**Authors:** Nur Sharmila Sharip, Hidayah Ariffin, Tengku Arisyah Tengku Yasim-Anuar, Yoshito Andou, Yuki Shirosaki, Mohammad Jawaid, Paridah Md Tahir, Nor Azowa Ibrahim

**Affiliations:** 1 Institute of Tropical Forestry and Forest Products (INTROP), Universiti Putra Malaysia, UPM Serdang, Selangor 43400, Malaysia; nursharmilasharip@gmail.com (N.S.S.); jawaid@upm.edu.my (M.J.); parida.introp@gmail.com (P.M.T.); 2 Department of Bioprocess Technology, Faculty of Biotechnology and Biomolecular Sciences, Universiti Putra Malaysia, UPM Serdang, Selangor 43400, Malaysia; tengkuarisyah@gmail.com; 3 Department of Biological Functions and Engineering, Graduate School of Life Science and Systems Engineering, Kyushu Institute of Technology, 2-4 Hibikino, Wakamatsu-ku, Kitakyushu, Fukuoka 808-0196, Japan; yando@life.kyutech.ac.jp; 4 Department of Applied Chemistry, Faculty of Engineering, Kyushu Institute of Technology, 1-1 Sensui-cho, Tobata-ku, Kitakyushu, Fukuoka 804-8550, Japan; yukis@che.kyutech.ac.jp; 5 Department of Chemistry, Faculty of Science, Universiti Putra Malaysia, UPM Serdang, Selangor 43400, Malaysia; norazowa@upm.edu.my

**Keywords:** ultra-high molecular weight polyethylene, cellulose nanofiber, bionanocomposite, melt-blending, ethanol mixing

## Abstract

The major hurdle in melt-processing of ultra-high molecular weight polyethylene (UHMWPE) nanocomposite lies on the high melt viscosity of the UHMWPE, which may contribute to poor dispersion and distribution of the nanofiller. In this study, UHMWPE/cellulose nanofiber (UHMWPE/CNF) bionanocomposites were prepared by two different blending methods: (i) melt blending at 150 °C in a triple screw kneading extruder, and (ii) non-melt blending by ethanol mixing at room temperature. Results showed that melt-processing of UHMWPE without CNF (MB-UHMWPE/0) exhibited an increment in yield strength and Young’s modulus by 15% and 25%, respectively, compared to the Neat-UHMWPE. Tensile strength was however reduced by almost half. Ethanol mixed sample without CNF (EM-UHMWPE/0) on the other hand showed slight decrement in all mechanical properties tested. At 0.5% CNF inclusion, the mechanical properties of melt-blended bionanocomposites (MB-UHMWPE/0.5) were improved as compared to Neat-UHMWPE. It was also found that the yield strength, elongation at break, Young’s modulus, toughness and crystallinity of MB-UHMWPE/0.5 were higher by 28%, 61%, 47%, 45% and 11%, respectively, as compared to the ethanol mixing sample (EM-UHMWPE/0.5). Despite the reduction in tensile strength of MB-UHMWPE/0.5, the value i.e., 28.4 ± 1.0 MPa surpassed the minimum requirement of standard specification for fabricated UHMWPE in surgical implant application. Overall, melt-blending processing is more suitable for the preparation of UHMWPE/CNF bionanocomposites as exhibited by their characteristics presented herein. A better mechanical interlocking between UHMWPE and CNF at high temperature mixing with kneading was evident through FE-SEM observation, explains the higher mechanical properties of MB-UHMWPE/0.5 as compared to EM-UHMWPE/0.5.

## 1. Introduction

Ultra-high molecular weight polyethylene (UHMWPE) is a long linear engineered thermoplastic with extremely high molecular weight of approximately 3 × 10^6^ g/mol [1]. It possesses high resistance against impact, fatigue, chemical corrosion and abrasion, which stemmed from effective load transfer to its long linear backbone. This polymer also has a remarkable self-lubricating, low friction coefficient and good biocompatibility [2,3,4] that enable its application in various fields including aerospace and industrial machineries (i.e., pipes, panels, bars, gears), microelectronics and joint replacement or also known as arthroplasty (i.e., hip liner, tibial inserts) [5,6,7]. However, relatively low Young’s modulus and surface hardness of UHMWPE could limit the sustainability of this polymer against wear as a result of contact and slip with harder counterpart such as metal under repeated motion [8]. This results in abrasion where generated debris in turns may accelerate cracks leading to component loosening and failure [4,5]. 

Various studies have been conducted involving fillers incorporation in UHMWPE matrix with the aim to improve its abrasion and wear through Young’s modulus enhancement. The fillers used ranged from inorganic to organic and natural fibers such as carbon nanofibers, hydroxyapatite as well as nanocellulose [9,10]. Besides improving the stiffness, the presence of fillers in polymer matrix could play a role in mitigating wear through its act as solid lubricant by rolling or sliding at interface between the contacted surfaces [11,12]. Nevertheless, this mechanism of solid lubrication is greatly dependent on the filler properties and size, where it could also become a third body abrasive that further abrade the UHMWPE surface, or further trigger the inflammation due to fillers cytotoxicity [10,13]. For instance, nanocellulose filler has been proven beneficial in enhancing wear resistance of UHMWPE and exhibit good biocompatibility against osteoblast cells MC3T3-E1 [5,11]. The nanocellulose debris was reported to serve as solid lubricant between metal and polymer surface, thus prevented further abrasion of UHMWPE, with relatively low wear volume as compared to neat UHMPWE. Additionally, nanocelluloses are biocompatible and non-toxic by which it can be used in many biomaterials application such as for wound dressings materials [14,15,16], scaffold for bone or tissue regeneration [17,18,19], carrier for drug deliveries [20,21,22,23,24] and many more [25,26,27,28]. These properties of nanocellulose make it an excellent material as UHMWPE fillers, particularly for artificial joint application.

Common method for nanocellulose composites fabrication in various matrices is through solution processing and melt blending, by which the latter is comparably easy, as well as industrially and economically viable [29,30,31]. In melt blending, nanocellulose is introduced and mixed with polymer in molten state [32,33,34]. Nonetheless, unlike most thermoplastic polymers, fabricating UHMWPE composites via conventional melt processing methods is extremely difficult. Viscous flow state of melt UHMWPE is not attainable even with increases in temperature, and it maintains in non-uniform or non-continuous rubberlike state. This is attributed by its higher theoretical viscous flow temperature as compared to its decomposition temperature [35], as a result of numerous chain entanglements contributed by its extremely high molecular weight. In fact, its melt viscosity could be up to 1 × 10^8^ Pa.s which is about 2500 times higher than high density polyethylene (HDPE) [36]. Similarly, UHMWPE composite fabrication by solution mixing is not convenient either, attributed to inertness of UHMWPE that is resilient to any reaction with acids, alkalis and organic solvent as well as biological reaction [37]. 

Wang et al. (2016) produced UHMWPE nanocellulose composites by mixing UHMWPE and nanocellulose in ethanol. The solution was continuously mixed until the ethanol was completely evaporated. This process aids in nanocellulose drying without the occurrence of aggregation up to 0.5 wt.% cellulose nanocrystals (CNC) loading. Nevertheless, results showed that better nanocellulose dispersion with higher micro-hardness was achieved through melt processing as compared to the ethanol mixing process. Yet, no information was given on the mechanical properties such as tensile strength, modulus and elongation of UHMWPE/CNC produced by the two different processes [11]. In our previous study, we fabricated UHMWPE/cellulose nanofiber (CNF) through melt blending process in triple screw kneading extruder. Despite homogenous filler dispersion and optimized parameters, the resulted tensile strength with 3 wt.% CNF loading was found decreased. In consideration that there is lack information on the mechanical properties of the UHMWPE/nanocellulose composites fabricated through different processing techniques, hence this study was conducted to investigate the effect of UHMWPE/CNF bionanocomposites blending process (melt and non-melt blending) on its mechanical and crystallinity properties. 

## 2. Materials and Methods 

### 2.1. Materials

Ultra-high molecular weight polyethylene (UHMWPE) were purchased from Sigma-Aldrich (ST. Louis, MO, USA) in the form of fine powder with particle size of 96 ± 20 µm. The molecular weight, melting point and density of the polymer was 3 × 10^6^–6 × 10^6^ g/mol, 138 °C and 0.94 g/mL, respectively. Meanwhile, 2 wt.% cellulose nanofiber (CNF) of 53.4 ± 9 nm diameter sizes was purchased from ZoepNano Sdn. Bhd. (Serdang, Malaysia) in slurry form. Absolute ethanol 99.8% AR grade was purchased from John Kollin Corporation (Midlothian, UK).

### 2.2. Bionanocomposite Fabrication and Moulding

**Figure 1:**
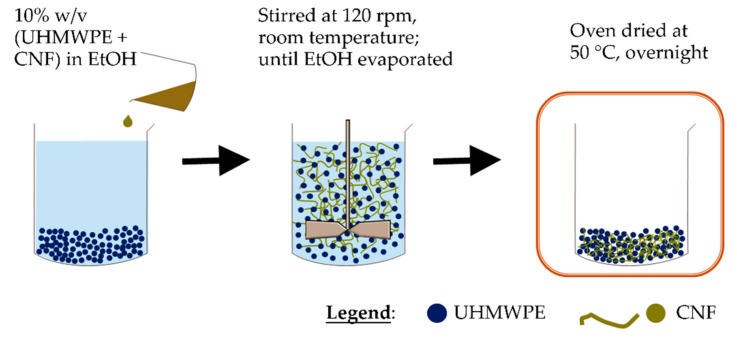
UHMWPE/CNF bionanocomposites by ethanol mixing.

Non melt-blending (ethanol mixing) process was conducted according to Wang et al. (2016) with some modification (Figure 1) [11]. About 10 wt. % UHMWPE-CNF (0.5 wt.% CNF in UHMWPE) was added into ethanol and mechanically stirred by using JLT Series Flocculators (Velp Scientifica, Usmate, Italy) at 120 rpm speed. The experiment was conducted at room temperature until the solvent was completely evaporated before being dried at temperature 50 °C overnight. 

For comparison, UHMWPE/CNF bionanocomposite of same composition was melt blended by using triple screw kneading extruder at Kyushu Institute of Technology, Fukuoka, Japan with optimized condition of 150 °C and 60 rpm rotational speed [38]. Meanwhile, UHMWPE without filler was subjected to both blending process and denoted as MB-UHMWPE/0 and EM-UHMWPE/0. The summary of produced bionanocomposites is as presented in Table 1. 

All samples were molded into 10 cm × 10 cm × 1 mm sheet by direct compression at 175 °C and 15 MPa for 45 min [39]. 

### 2.3. Characterization of Bionanocomposites

#### 2.3.1. Mechanical Analysis

The tensile properties was conducted by using a compact tensile and compression tester IMC-18E0 (Imoto Machinery Co., Ltd., Kyoto, Japan). Eight specimens of samples were subjected to a tensile tester with crosshead speed of 50 mm/min (ASTM D638).

#### 2.3.2. Morphological Analysis

The morpholocal analysis was carried out by using a high-resolution field-emission scanning electron microscopy (FESEM) (FEI Nova NanoSEM 230, FEI Company, Hillsboro, OR, USA) with accelerating voltage of 10 kV. Sample specimens subjected to tensile testing were analyzed for surface fracture and fiber matrix inter-relations. The tensile fractured samples were coated with platinum using a vacuum sputter coater prior to FESEM observation.

#### 2.3.3. X-Ray Diffraction Analysis

The crystallinity was measured by using a MiniFlex 600 X-ray diffractometer (XRD) (Rigaku Co., Tokyo, Japan) at 40 kV and 10 mA at room temperature. Cu Kα radiation (λ = 1.54 Å) was used as the X-ray source while the diffraction angle was scanned at 2θ from 3° to 50° at a rate of 20°/min. The crystallinity index (*CrI*) was calculated and determined based on this equation:

*CrI* = (*I*_total_ − *I*_am_)/*I*_total_ × 100 (1)

which *I_total_* and *I_am_* are the intensity of highest peak in crystalline region and amorphous region, respectively [40,41]. For example, in this study the *I_total_* for highest crystalline peak was approximately at 2θ = 22 while *I_am_* was at about 2θ = 21, representing the peak of the amorphous point.

### 2.4. Statistical Analysis

Statistical analysis was conducted by using Statistical Analysis Software (SAS^®^) University Edition through one-way ANOVA and Duncan’s Multiple range test at *p* < 0.05.

## 3. Results and Discussion

### 3.1. Mechanical Properties 

The mechanical properties of the polymers and the bionanocomposites samples are as presented in Table 2. The tensile strength of MB-UHMWPE/0 reduced by almost half of the value exhibited by Neat-UHMWPE while no significant difference was observed in EM-UHMWPE/0 sample. Yet, an opposite trend was observed when incorporating 0.5 wt.% CNF into the polymer matrix. Addition of CNF through ethanol mixing reduced the tensile strength by 34% from 55.4 MPa (EM-UHMWPE/0) to 36.6 MPa (EM-UHMWPE/0.5) whereas by melt blending, the reduction was only 11% which was from 31.8 MPa (MB-UHMWPE/0) to 28.4 MPa (MB-UHMWPE/0.5). Significant improvements in yield strength and Young’s modulus of MB-UHMWPE/0 sample by 15% and 25% were observed as compared to Neat-UHMWPE sample. These two mechanical parameters were also found increased in MB-UHMWPE/0.5 with 26% and 52% higher than Neat-UHMWPE. In other hand, yield strength, elongation, Young’s modulus and toughness of ethanol mixed samples were almost similar to Neat-UHMWPE except for elongation and toughness of EM-UHMWPE/0.5 that was 33% and 53% lower, respectively. On contrary, the toughness of melt-blended samples were largely affected by the process in which 48% and 31% lower value were obtained as compared to Neat-UHMWPE. Nevertheless, the toughness of MB-UHMWPE/0.5 (168.4 ± 3.2 J/m^3^) was 31% higher EM-UHMWPE/0.5 sample (116.5 ± 5.8 J/m^3^).

**Figure 2:**
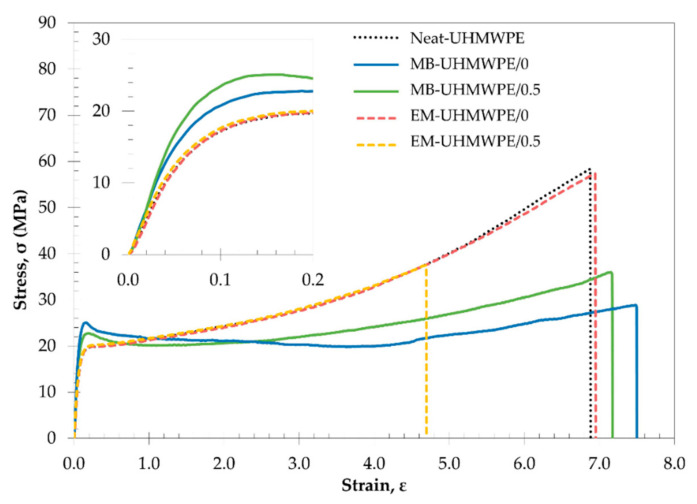
Representative engineering stress-strain curve of the neat UHMWPE and UHMWPE/CNF bionanocomposites showing differences in Young’s modulus (inlet with lower strain regime), yield strength and fracture strain.

The mechanical properties of the samples can be explained based on the representative stress-strain curve in Figure 2. Neat-UHMWPE exhibited a tough and ductile behavior, which was in agreement with published reports on UHMWPE characteristics [42,43,44,45]. An identical profile was observed on EM-UHMWPE/0 indicated that subjecting UHMWPE to ethanol at room temperature could less likely affect the UHMWPE polymer structure evident from minimal changes in mechanical properties and similar hardening or cold drawing behavior of the samples in uniaxial tension. This can be supported by published reports stated that UHMWPE is inert and resilient to any reaction with acids, alkalis and organic solvent as well as biological reaction [37,46,47]. The curves in Figure 2 also showed that the incorporation of filler through ethanol mixing was ineffective that the common filler stiffening effect in polymer matrix was not observed. On the contrary, processing through melt blending enabled significant improvement of yield strength and Young’s modulus which was very notable from the lower strain regime of stress-strain curves shown in the figure. It can be suggested that better UHMWPE-CNF adhesion was achieved through melt blending as compared to ethanol mixing. This is supported by published report stating that infiltration of melt polymer with addition of shear force during melt blending could results in smaller filler agglomerates and better interaction [48]. 

The information on toughness could also be obtained from the area under the stress-strain curve in Figure 2. As mentioned before, even though melt blending process reduced the toughness of the polymer (MB-UHMWPE/0 as compared to Neat-UHMWPE), the value when incorporating CNF (MB-UHMWPE/0.5) was apparently higher than the one produced through ethanol mixing (EM-UHMWPE/0.5). In respect to their respective polymer subjected to the same blending process without addition of CNF (MB-UHMWPE/0 and EM-UHMWPE/0), melt blending process enabled improvement in toughness of MB-UHMWPE/0.5 sample by 25%, whereas reduction by 48% was observed on ethanol mixed bionanocomposite sample (EM-UHMWPE/0.5). This proved the efficiency of melt blending for producing UHMWPE/CNF bionanocomposites as penetration of molten polymers into the fillers during high temperature processing could results in better mechanical interlock between the matrix and filler even without any chemical bonding presence [49,50]. 

**Figure 3:**
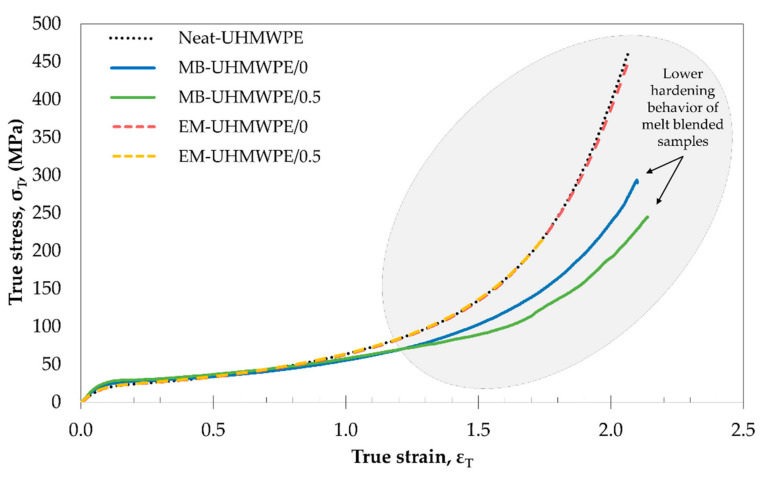
The true stress-strain curve of the neat UHMWPE and UHMWPE/CNF bionanocomposites showing hardening or cold drawing portion behavior in uniaxial tension.

In term of tensile strength, the reduced value in melt blending samples could be attributed to some molecular weight reduction and chain scission during melt processing. Although UHMWPE is thermally stable up to 400 °C, chain scission of the polymer may occur at lower temperature due to mechanically-initiated breaks such as shear forces [51,52,53], which is very likely to occur in melt blending. However, recrystallization of newly formed shorter chain could contribute to increased crystallinity and toughness besides enhancing diffusion of polymer for improved chain entanglement [54,55,56], providing better intrinsic properties of the composites. This was confirmed by the hardening behavior of samples as presented in Figure 3. Both melt blended samples (MB-UHMWPE/0 and MB-UHMWPE/0.5) exhibited lower hardening profile as compared to Neat-UHMPWE and ethanol mixed (EM-UHMPW/0 and EM-UHMWPE/0.5). According to Kurtz (2016), the hardening behavior of UHMWPE is sensitive to its molecular weight in which lower molecular weight exhibited lower hardening profile and vice versa [57,58]. Albeit this, the tensile strength of the CNF-incorporated melt blended sample (MB-UHMWPE/0.5) differed by only 8 MPa as compared to the one produced through ethanol mixing (EM-UHMWPE/0.5). It is also important to note that the tensile strength of MB-UHMWPE/0.5 sample which was 28.4 ± 1.0 MPa surpassed the minimum requirement of standard specification for fabricated UHMWPE for surgical implant, ASTM F648-14 which is 27 MPa [59]. 

### 3.2. Morphological Properties

**Figure 4:**
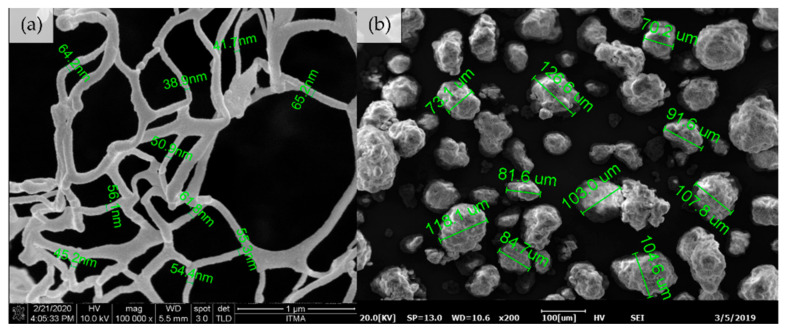
Scanning electron microscope images of (**a**) CNF and (**b**) UHMWPE.

The morphology of CNF and UHMWPE was observed under 1 × 10^5^ and 200 times magnification using scanning electron microscope, accordingly (Figure 4). The average diameter for single nanocellulose fiber and UHMWPE resin was about 53 ± 9 nm and 96 ± 20 µm, respectively. 

**Figure 5:**
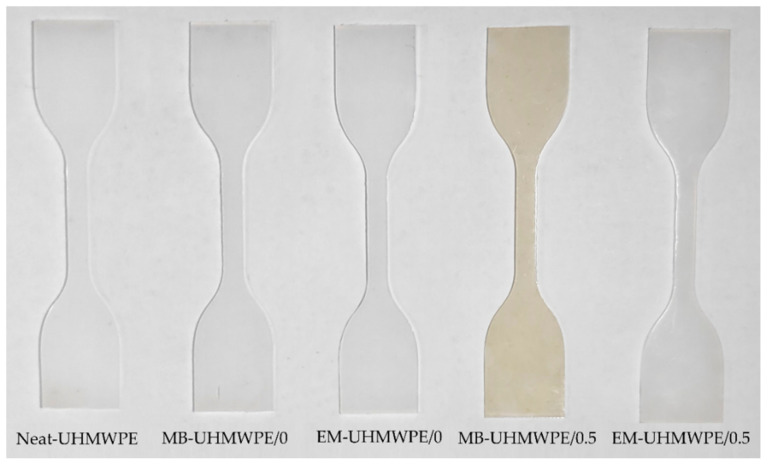
The visual appearance of polymer and UHMWPE/CNF bionanocomposites samples produced through melt blending and ethanol mixing.

Meanwhile, the Neat-UHMWPE film appeared white and subjecting the polymer to different blending process did not affect its color appearance (Figure 5). The absence of color changes in melt blended polymer without CNF (MB-UHMWPE/0) proved that processing the polymer (with combination of heat and mechanical stress) at temperature 150 °C did not lead to UHMWPE thermal decomposition. Absence of notable changes on the color appearance was also observed with incorporation of 0.5% CNF via ethanol mixing (EM-UHMWPE/0.5). In the meantime, processing the bionanocomposites with 0.5 wt.% CNF through melt blending resulted in yellowish MB-UHMWPE/0.5 sample, suggesting some effect of heat degradation on CNF. A combination of heat and mechanical force exerted on CNF during melt blending might be a contributing factor thus explained the different in MB-UHMWPE/0.5 appearance regardless of same amount of filler loading with EM-UHMWPE/0.5. According to Heggset et al. (2017) [60], one of indicators for nanocellulose decomposition at high temperature was color changes in which the percentage of color changes increased with increased of temperature (110 °C to 150 °C). The appearance of yellow/brownish/black from colorless/white was deemed associated to thermal oxidation in the presence of oxygen. In oxidation and hydrolysis reactions, aldehyde and carboxyl groups were formed and the resulted carbonyl group generated in the cellulose chains influenced its color appearance [61,62]. Formation of furan type compounds in thermal degradation of carbohydrates was also a responsible factor for the color changes in cellulose due to high temperature [63,64].

**Figure 6:**
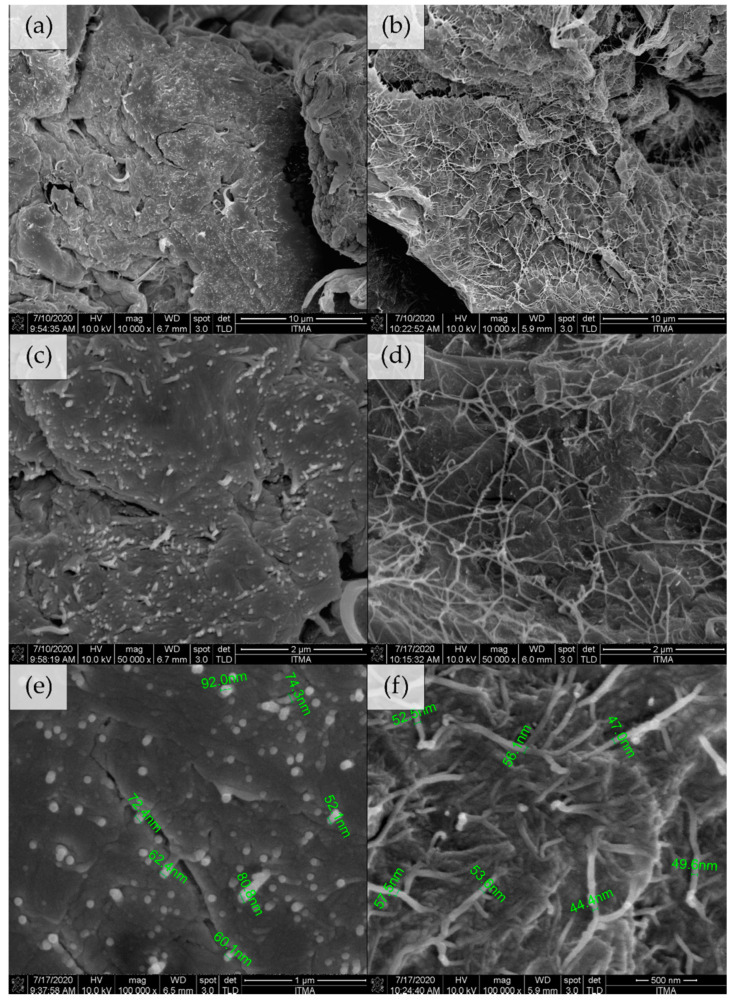
Fracture surface of UHMWPE/CNF prepared by (**a**,**c**,**e**) melt blending and (**b**,**d**,**f**) ethanol mixing at 10,000×, 50,000× and 100,000× magnification, respectively.

The fractured section of the MB-UHMWPE/0.5 and EM-UHMWPE/0.5 samples were observed to investigate the configuration of CNF filler in UHMWPE matrix by both processes. It is important to note that the diameter of CNF in MB-UHMWPE/0.5 increased to 71 ± 14 nm (Figure 6e) from 53.4 ± 9 nm of its initial size (Figure 4a), whereas the diameter of CNF in EM-UHMWPE/0.5 sample was about the same (52 ± 5 nm) (Figure 6f). Increased in CNF diameter size by melt blending was attributed to the CNF fast drying in triple screw kneading extruder aided by high temperature processing. Meanwhile, a notable appearance of mesh-like CNF can be observed covering the fractured polymer of EM-UHMWPE/0.5 sample (Figure 6b,d). In comparison, CNF in MB-UHMWPE/0.5 were embedded and fractured along with the polymer (Figure 6a,c). 

**Figure 7:**
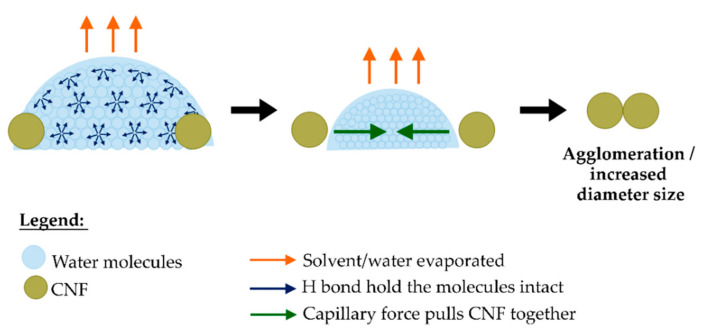
Schematic representation of CNF drying in bionanocomposites blending process.

The explanation to the increased diameter of CNF during drying can be shown by the schematic representation in Figure 7, whereby it is shown that the removal of water molecules during drying leads to the formation of capillary forces exerted on the hydrophilic cellulose. This capillary effect causes the adjacent fibers to be drawn together and formed strong hydrogen bonding and hence, caused increase in diameter size [65,66,67]. Capillary tension increases with the increase in vapor pressure, which is affected by the temperature increment [68,69,70]. Processing bionanocomposites through melt blending at high temperature (150 °C) caused the increment in capillary tension resulting in bigger CNF diameter size as compared to the one processed through ethanol mixing at room temperature. Even though CNF drying was also occurred in ethanol mixing, the presence of alcohol reduced the interfacial tension of the liquid-water interface. This was due to the disruption of hydrogen bond network corresponded to the decrease of water-water hydrogen bond [71]. Ethanol also possesses lower surface tension at 25 °C (22 × 10^−3^ J/m^2^) which is much lower than water at higher temperature of 100 °C (58.9 × 10^−3^ J/m^2^) [72]. The use of alcohols such as ethanol, methanol and butanol in nanocellulose drying provides more interfibrillar distance than water only due to their higher molecular size as compared to water molecules. This is beneficial in reducing interfibrillar contacts and adhesion between nanocellulose fibers [73,74]. 

**Figure 8:**
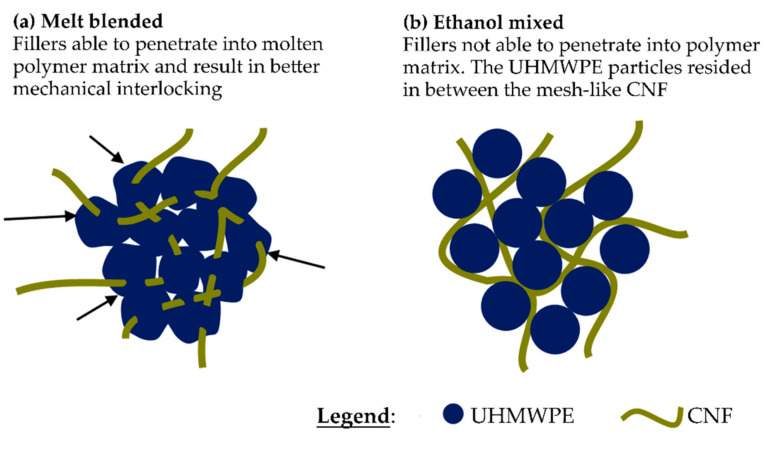
Illustration of CNF interaction with UHMWPE matrix in (**a**) melt blended (MB-UHMWPE/0.5) and (**b**) ethanol mixed (EM-UHMWPE/0.5) samples.

Mesh-like observations of the CNF in UHMWPE matrix bionanocomposites can be schematically viewed in Figure 8. The melt blending process enables mixing of CNF in molten state of UHMWPE thus allowing penetration of filler into matrix particle (Figure 8a). The penetration of filler into matrix and the shrinkage of the polymer during cooling developed mechanical interlocking between filler and matrix when molded, thus resulted in better mechanical properties [49,75]. Through ethanol mixing method, the CNF could not penetrate into the non-molten UHMWPE matrix. Instead, the UHMWPE particles resided in between the mesh-like CNF (Figure 8b) and combined through continuous mixing whilst solvent evaporated.

### 3.3. Crystallinity Evaluation

**Figure 9:**
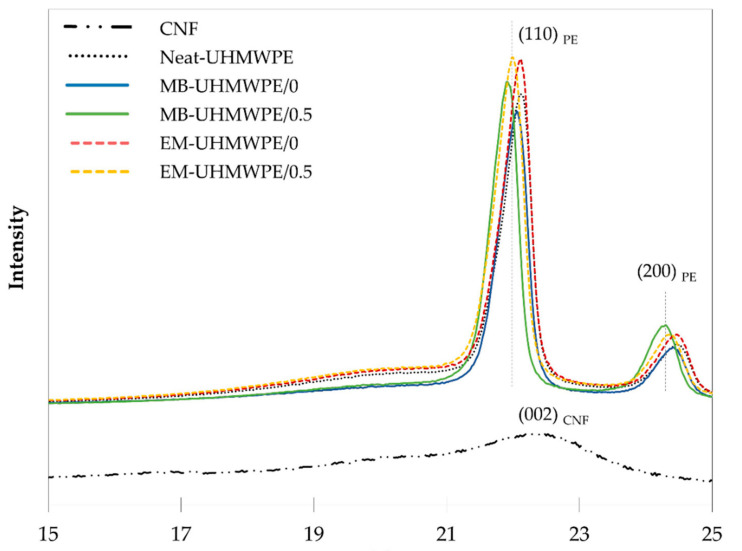
X-ray diffractogram of UHMWPE/CNF bionanocomposites.

The x-ray diffraction pattern of UHMWPE and CNF are presented in Figure 9. The calculated crystallinity index of the two materials in respect to their respective total peak height were 78% and 50%, in the same order. The UHMWPE peak was seen to exhibit sharp increase of crystalline peak befitted long and unbranched polymer crystalline structure and arrangement [76,77], while CNF peak shows semi crystalline pattern indicating existence of crystalline and amorphous region of the cellulose chains [78,79]. Additionally, all polymer and bionanocomposites samples exhibited similar pattern with two prominent diffraction peaks centered at around 22.0° and 24.4° of 2θ, which correspond to (110) and (200) reflection of polyethylene in orthorhombic phase [80,81,82]. The diffraction peak of the filler could not be observed due to low percent loading [81,83] and overlapped peak of UHMWPE with CNF at around 22° in 2θ, which was in agreement with reported studies involving nanocellulose filler in polyethylene matrix [40,84]. Reduction in amorphous region was observed between 18° to 21° for samples fabricated through melt blending suggesting an improvement in chain entanglement and improved crystallinity stemmed from chain scission occurrence [54,55]. 

MB-UHMWPE/0 and EM-UHMWPE/0 had crystallinity of 83% and 78, respectively. When incorporated with 0.5% CNF, the melt-blended MB-UHMWPE/0.5 had a slight increment in crystallinity to 86%, while the crystallinity of ethanol-mixed EM-UHMWPE/0.5 remained. The increment of crystallinity for melt-blended samples with and without CNF is in correlation with the possible occurrence of polymer chain scission. Even in temperature lower than its decomposition temperature, chain scissioning is possible due to mechanically initiated breaks caused by other factors including shear forces [51,52,53]. According to Fu and co-workers, chain scissioning of UHMWPE lead to recrystallization of newly formed shorter chain hence contributed to increased [54,55,56]. The formation of shorter chain also enhanced the diffusion of polymer and improved the chain entanglement resulting in higher crystallinity. On another note, the addition of cellulose nanomaterials also may act as nucleating agent, which has been previously [30,40,85,86,87] As a nucleating agent, CNF as natural fiber could induce more formation of crystallites in the polymer matrix [29]. 

## 4. Conclusions

Comparison in mechanical properties of UHMWPE/CNF bionanocomposites fabricated through non-melt blending (ethanol mixing) and melt blending process revealed that bionanocomposites from the latter method had better properties. Significantly higher yield strength, elongation at break, Young’s modulus, toughness and crystallinity by 28%, 61%, 47%, 45% and 11%, respectively, were achieved through melt blending as compared to ethanol mixing. A better mechanical interlocking between UHMWPE and CNF was seen through FE-SEM micrographs indicating a good blending of CNF with the polymer matrix, assisted by the use of elevated temperature and kneading. Lower tensile strength (22%) of melt-blended UHMWPE/CNF was recorded, indicating the occurrence of chain-scission during melt blending as evidenced by the reduction in strain hardening. Nevertheless, the tensile strength value surpassed the minimum requirement of standard specification for fabricated UHMWPE in surgical implant application. The results demonstrated melt blending as a better fabrication process for producing UHMWPE/CNF compared to ethanol mixing, with the advantage of being easily scalable for larger scale processing. 

## Figures and Tables

**Table 1:** UHMWPE/CNF bionanocomposite samples and control.

Sample	Blending Process	CNF Content (wt.%)	Remarks
Neat-UHMWPE	none	0	control for blending effect
MB-UHMWPE/0	Melt blending	0	control for CNF addition effect by melt blending
MB-UHMWPE/0.5	Melt blending	0.5	-
EM-UHMWPE/0	Ethanol mixing	0	control for CNF addition effect by ethanol mixing
EM-UHMWPE/0.5	Ethanol mixing	0.5	-

CNF: cellulose nanofiber, UHMWPE: ultra-high molecular weight polyethylene, MB: melt blend, EM: ethanol mixed.

**Table 2:** Effect of blending process and CNF addition on the mechanical properties of UHMWPE.

	Neat UHMWPE Properties	Mechanical Properties (% Difference Compared to Neat-UHMWPE)			
		MB-UHMWPE/0	MB-UHMWPE/0.5	EM-UHMWPE/0	EM-UHMWPE/0.5
Tensile strength (MPa)	62.1 ± 5.0	31.8 ± 3.1 * (−49)	28.4 ± 1.2 * (−54)	55.4 ± 3.3 (−11)	36.6 ± 1.1 * (−41)
Yield strength (MPa)	20.6 ± 0.6	23.8 ± 0.8 * (+15)	25.9 ± 0.5 * (+26)	20.0 ± 0.2 (−3)	20.3 ± 0.1 (−2)
Elongation (%)	691.1 ± 37.4	726.0 ± 45.2 (+5)	749.6 ± 2.0 * (+8)	694.2 ± 40.6 (0)	465.1 ± 4.2 * (−33)
Young’s modulus (MPa)	267.9 ± 22.4	334.3 ± 14.4 * (+25)	407.9 ± 24.3 * (+52)	254.1 ± 17.4 (−5)	276.8 ± 0.2 (+3)
Toughness (J/m^3^)	245.7 ± 21.3	128.8 ± 40.7 * (−48)	168.4 ± 3.2 * (−31)	224.3 ± 17.3 (−9)	116.5 ± 5.8 * (−53)

MB: melt blend, EM: ethanol mixed, UHMWPE: ultra-high molecular weight polyethylene. Asterisk (*) indicates significant difference of samples with neat UHMWPE (*p* < 0.05).
